# Socio-Demographics of Initial Substance Use Exposure and Its Relation to Progression: A Cross-Sectional Study in Saudi Arabia

**DOI:** 10.7759/cureus.42795

**Published:** 2023-08-01

**Authors:** Alaa Nabil Mahsoon, Lina Almashat, Norah Alsubaui, Shahad Hindi, Shahad Alharbi, Sara Yaghmour, Loujain Sharif

**Affiliations:** 1 Department of Psychiatric and Mental Health Nursing, Faculty of Nursing, King Abdulaziz University, Jeddah, SAU; 2 Faculty of Nursing, King Abdulaziz University, Jeddah, SAU

**Keywords:** initial exposure, substance users, substance abuse, saudi arabia, socio-demographics

## Abstract

Introduction: Empirical evidence on substance use in Saudi Arabia is lacking. This quantitative study is aimed at describing the socio-demographics of initial exposure to substance use and its relation to substance abuse progression.

Method: A questionnaire about socio-demographics during initial exposure to substance use was completed by 379 participants.

Results: For most participants, the commencement of substance abuse occurred at the age range of 19‒23 years, and while in high school, they first started taking drugs with school friends. The two psychoactive substances most commonly taken for the first time were hashish and alcohol. The two main reasons for first drug exposure were teenage curiosity and joy-seeking. The chi-square test revealed statistically significant differences between substance abuse progression by sex, current age, father’s education level, parent’s marital status, and one’s company in substance use. Female participants were more likely to continue taking drugs.

Conclusion: Young people must be educated about the risks and consequences of substance use from early adolescence.

## Introduction

Drug abuse is a severe and rapidly spreading problem globally. The World Drug Report by the United Nations Office on Drugs and Crime defines substance abuse as the continued use of psychoactive substances such as alcohol and illicit drugs in a harmful or risky manner [1]. The psychological, behavioral, and physical symptoms of substance abuse negatively affect a person’s quality of life, family members, and society. Substance use is among the top 20 risk factors for poor health worldwide [2]. Nevertheless, statistics on substance abuse are low in Saudi Arabia, which may be linked to the nation’s societal norms and values deeply influenced by religion as an Islamic country. While it is religiously and legally prohibited to manufacture, sell, possess, or consume alcohol and narcotic substances, Saudi Arabians consume alcohol and use drugs [3,4]. Despite the widespread use in the Middle East, limited studies have investigated substance use in Saudi Arabia, and none have addressed in depth the initiation of drug use in the nation. Saquib et al. reported that socio-demographic data related to the drug initiation experience, including family influences, religion, age at first use, media, peer pressure, and drug culture, are lacking [4]. Therefore, the possible relation of specific cultural socio-demographics to the initial exposure and progression to substance use in Saudi Arabia should be investigated.

Although substance abuse is a health problem that is preventable and treatable through effective, comprehensive, and multidisciplinary intervention, it remains among the world’s most severe and rapidly expanding issues. According to the World Drug Report by the United Nations Office on Drugs and Crime 2020 [1], thousands of people worldwide abuse psychoactive substances; further, the prevalence of substance abuse increased by more than 12% between 2009 and 2018. Despite the aforementioned factors related to the limited substance abuse data in the country, Saudi Arabia is more prone to illicit abuse than previously believed [5]. An increase has also been observed in the number of drug and alcohol abuse treatment centers in Saudi Arabia [6]. Among the Saudi population and worldwide, the most common substances abused are cannabis, amphetamines, heroin, alcohol, and stimulants [2].

The impact of substance abuse on human life is characterized by mental, behavioral, and physical symptoms, whether the drug is taken for recreation or on prescription. For example, excessive use can increase the risk of health problems such as injuries, motor vehicle accidents, violence, neurological syndromes, and liver cirrhosis [1]. Furthermore, younger individuals have a higher risk of developing health problems from substance use disorders than older adults [7], with cannabis being the most popular drug among these users [8]. Health problems are not the only consequences of substance abuse; economic, societal, and legal problems are also linked to substance abuse [9].

Individuals engage in substance use for diverse reasons, and young people more commonly use illicit drugs than older people [10]. A combination of situations and factors influence young adults’ susceptibility to drug abuse while also developing their personalities during this developmental stage; these involve low self-esteem, the need to gain acceptance from peers, and the desire for societal admiration [11]. In addition, drugs are abused to lower anxiety and raise concentration during stressful times, such as exams [12]. A study that included 112 participants undergoing detoxification therapy for opiate usage indicated that most impacted respondents had difficulties managing their emotions [13]. A systematic review showed that the risk factors of substance use engagement could be at individual, family, and community levels [10]. The risk factors at the individual level included easy access to drugs, emotional problems, impulsivity traits, not being strongly religious, and psychological trauma and disorders. The risk factors at the family and community levels involved low parental education, neglect, lack of control, and mimicking abusive behaviors exhibited by their parents or peers [10].

Socio-demographic characteristics are central in the assessment of an individual’s substance use experience. Only a few studies have attempted to describe the related socio-demographic characteristics of the Saudi population [4]. Saudi Arabians with substance abuse issues are typically considered to be young, unemployed men with low educational status [14]. A study conducted in the Al-Qassim region, Saudi Arabia, including 612 substance abusers, showed that 64.5% of abusers were high school students and were polysubstance abusers (41.9%) [2]. In addition, an earlier study conducted in Saudi Arabia reported that the mean age of substance abusers was 29 years, with those who abuse heroin being younger than those who use only alcohol [15]. Moreover, demographic factors, such as being unemployed, single, middle-aged (for opium dependence), and young (for cannabis and stimulant abuse), are associated with substance-use dependence [6]. However, in the Saudi cultural context, essential socio-demographic characteristics need to be considered, for example, religion and family, as they play a crucial role in the Saudi culture and may contribute to a deeper understanding of substance use engagement. Nevertheless, most prior studies that described the demographic characteristics of substance abuse did not consider aspects such as the family’s religious background and that Saudi families tend to be large, sometimes with multiple wives in the family [4]. Moreover, most studies focused on an individual's current substance use status; to the best of our knowledge, no studies have explored the status of the socio-demographics of individuals when they first engaged in substance use. A study investigated the stages of progression in drug involvement among ten adolescents and 91 adults in Saudi Arabia and found that intercultural differences are likely in the initiation stages [5]. Therefore, data on the first socio-demographic characteristics specific to Saudi Arabia's culture are needed to understand better how characteristics might be involved with the initiation and progression of substance use. This study aims to describe the demographics of the initial exposure to substance use and its relation to substance abuse progression in the Saudi context.

## Materials and methods

Study design

This study was exploratory, and since little is known about this topic, a quantitative, descriptive, correlational, and cross-sectional design was implemented. The descriptive design defines a population, condition, or event being studied accurately and systematically [16]. A correlation design is used to investigate the interrelationships between variables of interest without the involvement or implementation of a researcher [16]. Moreover, a cross-sectional study is designed to collect information from people at a single time point [17]. This research design was chosen to describe the socio-demographics of individuals' initial exposure to substance use and its relation to substance abuse progression.

Setting

This study was conducted in affiliation with the Faculty of Nursing at King Abdulaziz University, Jeddah, Saudi Arabia. However, the data were obtained from the general population of Saudi Arabia via Google Forms.

Sample size

Because of the study’s descriptive nature, the convenience sampling technique was preferred. A large sample size was desired to determine the socio-demographic characteristics of the initial exposure to substance use. However, since no prior data on the estimated number of Saudi individuals with substance abuse could be found, an estimated sample size based on psychometric analysis was calculated using the Raosoft program [18]. The minimum sample size needed for the study was n=377, with a confidence interval of 95% and a margin error of 5%. The psychometric analyses utilized for the correlation analysis were two-tailed tests at a 0.05 level of significance and power of 0.80 with a medium effect size of 0.3.

Study population

Participants were substance users recruited from the public. Inclusion criteria were (a) engagement with substance use and (b) being able to read English and Arabic. 

Study duration

Researchers collected the data immediately after obtaining approval from the ethical committee of the Nursing Research and Ethics Committee. The study was conducted over four months.

Data collection

Socio-demographic data included questions about when the participants first engaged in substance use. The questions sought the following details: age, family size, marital status, educational level, parents’ social status, number of fathers’ wives, parents’ educational level, parents’ occupations, socioeconomic class, and the family’s and individual’s religious status. In addition, data on how the individual first knew about the drug, who the individual first used the drug with, the name of the first drug used, and the reason for starting drug use were collected with a questionnaire survey. The progression of substance abuse was addressed with a one-item question: "Do you still use drugs/alcohol?" (Appendices).

The researchers developed the demographic questionnaire after intensively reviewing the literature on this topic [19]. Socio-demographic data included questions about when the participants first started engaging in substance use. The questions included the following: age, gender, family size, marital status, educational level, parent's social status, number of fathers' wives, parent's educational level, parent's occupations, socioeconomic class, and the religious status of the family and the individual. In addition, data on how the individual first knew about the drug, whom the individual first used the drug with, the name of the first drug used, and the reason for starting using were addressed in the questionnaire as well. The progression of substance abuse was addressed with one item question that is "Do you still continue using drugs/alcohol?" (Appendices).

The demographic data in the questionnaire underwent face validity by seven experts in psychiatric mental health, who work as professors at the Department of Psychiatric and Mental Health Nursing at King Abdulaziz University. The experts reviewed and approved the tool. In this study, only data related to the socio-demographic characteristics during initial substance-use exposure were collected. Hence, the reliability test was not applicable as no tools were used to measure the variables.

In this study, the primary data were collected using a questionnaire. After obtaining the necessary ethical approval to conduct the study, the questionnaire was distributed in English and Arabic languages using an online survey tool. The participants were approached via social media platforms such as WhatsApp, Twitter, and Facebook. The participants were asked to distribute the link to increase the number of study participants. The participants were informed about the inclusion criteria at the beginning of the survey to ensure they met the required criteria.

Data analysis

Data were analyzed using SPSS Statistics for Windows, Version 26.0 (IBM Corp, Armonk, NY). Descriptive statistics included frequencies and percentages to describe the categorical variables of the socio-demographic data. For inferential statistics, the chi-square test was used to test the significance of differences among categorical variables. The Spearman test was applied to determine the correlation between the progression of substance use and the socio-demographics pertaining to first substance use.

Ethical considerations

Ethical approval for conducting the study was obtained from the Nursing Research and Ethics Committee (NREC Serial No: Ref No 2B.22).

Data collection was completely anonymous, and enrolment in the study was voluntary; apart from the socio-demographic details sought in the questionnaire, no other personal information was requested. On the first page of the online survey tool, detailed information about the study was presented. Hence, before the participants started filling out the survey, they had the opportunity to read the information provided and decide whether to engage in the study. Informed consent was obtained from participants completing and submitting the survey.

## Results

Sample characteristics

Of the 400 questionnaires shared among the respondents, 379 were returned (response rate: 94.75%).

Gender: The outcomes from the respondents suggest that early substance use exposure was more common among males (76.52%) than females. However, females tend to have longer addiction periods than males altogether.

Age: Based on the cohort selected for the study, it was determined that early exposure to substance use occurred much later in life. The age at which most respondents commenced substance abuse was 19‒23 years, followed by 13‒18 years, and drug use started while in high school. Approximately half of the participants started taking drugs with their school friends (53.83%).

Education: Early exposure to substance use was also linked to the level of education and subsequent importance placed on education, particularly the parents or guardians. Both parents of most respondents had attained school-level education (38.52% of fathers and 37.99% of mothers).

Socioeconomic status (SES): In most cases, during first exposure, the participants were from the middle socioeconomic class (62%), had moderate-sized families (43%), and had their parents living together (70.89%). Further, most of the participants’ fathers were employed (54.09%), and their mothers were unemployed (62.53%).

Religious status: Most participants’ family religion varied between lenient and strict (61.21%).

Most used drugs: Three drugs that were first taken by most of the participants were hashish (53%), alcohol (42%), and amphetamine (lajjah) (33%).

The three main reasons identified for participants’ first exposure to drugs were teenagers’ curiosity (56%), joy-seeking (47%), and having free time (22%). In addition, 29% of the participants (42.7% female and 57.3% male) responded that they continued using drugs (Table 1)

**Table 1:** Socio-demographic characteristics of the participants (n=379)

Items	Frequency	%
Gender		
Male	290	76.52
Female	89	23.48
Total	379	100.00
Age (years)		
Less than 18	7	1.85
18–22	64	16.89
22–26	90	23.75
26–30	73	19.26
More than 30	145	38.26
Total	379	100.00
Family size when you first started using drugs/alcohol		
3	111	29.29
4	42	11.08
5	60	15.83
More than 6	166	43.80
Total	379	100.00
Marital status when you first started using drugs/alcohol		
Single	316	83.38
Divorced	17	4.49
Married	46	12.14
Total	379	100.00
What was your educational status when you first started using drugs/alcohol?		
Primary school	10	2.64
Middle school	51	13.46
High school	160	42.22
College	150	39.58
Not educated	8	2.11
Total	379	100.00
What was your parents’ marital status when you first started using drugs/alcohol?		
Together	269	70.98
Separated	27	7.12
Divorced	34	8.97
Widowed	49	12.93
Total	379	100.00
How many wives did your father have when you first started using drugs/alcohol?		
0	93	24.54
1	196	51.72
2	66	17.41
3	13	3.43
4	11	2.90
Total	379	100.00
How did you first know about/who did you first use drugs/alcohol with?		
Parents	8	2.11
Siblings	5	1.32
Cousins	40	10.55
School friends	204	53.83
Friends outside school	78	20.58
Husband or fiancé	8	2.11
Girlfriends or boyfriends	16	4.22
Total	359	94.72
Missing	20	5.28
Total	379	100.00
Father's educational level when you first started using drugs/alcohol		
School	146	38.52
College	105	27.70
Higher education	37	9.76
None	91	24.01
Total	379	100.00
Mother's educational level when you first started using drugs/alcohol		
School	144	37.99
College	82	21.64
Higher education	23	6.07
None	130	34.30
Total	379	100.00
What was the status of your father's occupation when you first started using drugs/alcohol?		
Not employed	52	13.72
Employed	205	54.09
Retired	82	21.64
Not applicable	40	10.55
Total	379	100.00
What was the status of your mother's occupation when you first started using drugs/alcohol?		
Not employed	237	62.53
Employed	78	20.58
Retired	17	4.49
Not applicable	47	12.40
Total	379	100.00
What was your socioeconomic class when you first started using drugs/alcohol?		
Low	47	12.40
Middle	236	62.27
High-middle	81	21.37
High	15	3.96
Total	379	100.00
How would you describe the religious status of your family when you first started using drugs/alcohol?		
Strict	51	13.46
Between strict and lenient	232	61.21
Lenient	86	22.69
Not religious	10	2.64
Total	379	100.00
What is the name of the drug you used for the first time?		
Xanax (benzodiazepine)	46	0.13
Amphetamine (lajjah)	122	0.33
Hashish	194	0.53
Crystal myth	24	0.07
Ecstasy	10	0.03
Morphine	13	0.04
Heroin	10	0.03
Gat/Qat (*Catha edulis*)	42	0.11
Shammah (chewing tobacco)	44	0.12
Alcohol	152	0.42
LSD	12	0.03
Lyrica	46	0.13
Total	715	1.95
What was your age when you first started using drugs/alcohol (years)?		
12 or below	13	3.43
13–18	112	29.55
19–23	154	40.63
24–29	77	20.32
30–35	15	3.96
Above 35	8	2.11
Total	379	100.00
What was the first reason that you started using drugs/alcohol for?		
Teenagers’ curiosity	210	0.56
Joy-seeking	174	0.47
Psychological disorder	53	0.14
Lack of knowledge about complications of drugs	68	0.18
Positive attitude toward drug abuse	23	0.06
Low self-confidence	38	0.10
To eliminate shyness	25	0.07
Lack of amusement facilities	37	0.10
Disability in resolving routine problems	25	0.07
Crowded family	35	0.09
Having strict parents	24	0.06
Access to drugs	63	0.17
Lack of access to consultation centers	12	0.03
Low cost of drugs	20	0.05
Having free time	81	0.22
Presence of an addicted person in residential/educational place	28	0.08
Total	916	2.46
Do you continue to use drugs/alcohol?		
Yes	110	29.02
No	269	70.98
Total	379	100.00

Correlation between participant socio-demographics at initial exposure and substance abuse progression

The chi-square test revealed statistically significant differences between substance abuse progression and the following variables: gender (p=0.000), current age (p=0.008), father’s education level (p=0.042), parents’ marital status (p=0.022), and one’s company in substance use (p=0.002).

For correlation analysis, Spearman’s test revealed only one association between gender and substance abuse progression (Spearman’s value=-0.290, p=0.000); the results indicated a significant negative correlation between the variables, suggesting that females are more likely to continue taking drugs. None of the other variables showed significant correlations.

## Discussion

Our study results showed that most users were male (76.52%). Despite female participants being in the minority in drug and substance abuse in this study, they tended to use substances longer than male participants. In a study of 207 female patients undergoing treatment and rehabilitation for substance abuse, the participants claimed that Saudi Arabia’s strict and conservative nature meant women had less access to drugs than men [20]. However, while Saudi Arabian women might find it difficult to access drugs, they could be distressed when given drugs to treat mental health issues. A case study of patients undergoing rehabilitation at Al-Amal Rehabilitation Centre indicated that most female participants’ substance use was triggered by their vulnerability and adverse experiences, such as past incidences of sexual abuse, rape, unstable marriages, and suicidal thoughts [20]. Thus, we infer that while women might not be easily exposed to drugs initially, once they have access to substances and commence drug use, they may find it difficult to quit and continue using drugs for a long time. Moreover, an existing study reported that gender differences significantly influence increased susceptibility to drug use and relapse; specifically, compared with men, women become more compulsive drug users [21].

In our study, we found that the age range most vulnerable to the commencement of substance abuse was 19‒23 years, followed by 13‒18 years, and drug use started while in high school. Further, most participants reported being accompanied by school friends during initial drug use. The outcome could be related to life experiences or circumstances, with social group relations or associations potentially mediating later life outcomes or predispositions towards substance use and abuse. A comparison with several other studies indicated that most users experiment with drugs during adolescence [22]. Most studies pinpoint that the dramatic spurts of both physical and intellectual growth occurring during adolescence make teenagers develop an experimental mindset and become more prone to drug use [23].

While not directly measured in the study, the analysis included critical socioeconomic status determinants. The first was education, commonly associated with improved SES outcomes. The inference is that an improved SES potential can lead to a generally healthy lifestyle through access to health and well-being resources like healthcare and support networks, reducing the potential for substance abuse. A study on attitudes toward illicit drug abuse and correlated demographics conducted on 316 healthcare students in Saudi Arabia, in which the fathers of more than half of the participants had university-level education, found that fathers’ education level was significantly associated with the mean attitude score [24]. Another study on parental influence on substance use conducted in the USA indicated that parents’ level of education was a determining factor in their communication and understanding of teenagers, which consequently impacted their exposure to substance abuse [25]. However, life circumstances and conditions and the benefit of resource access to facilitate substance use can be considered a downside to SES.

The study managed to study the relationship between marriage and substance use exposure and progression. Among the participants, 83.38% reported being single when they first started using drugs, 12.14% were married, and 4.49% were divorced. Idleness and other external influences, such as having fewer restrictions on freedom than those married, might influence unmarried individuals. These suggest that marriage and involvement with family or household development tend to reduce proclivities towards substance use and abuse, with single individuals with a high level of SES more likely to experience addiction. Idleness and other external influences, such as having fewer restrictions on freedom than those married, might influence unmarried individuals. In a study conducted in Dammam, 12,743 patients with substance abuse issues (60%) were never married. Being single may influence continued heavy alcohol use and moderate and heavy marijuana use [26].

In addition, religious beliefs as a determinant of knowledge and insight generation among children were also vital to understanding early exposure to substance abuse and subsequent progression. In this respect, most respondents were raised in families classified as strict and lenient. There was a higher predisposition towards substance use from children hailing from lenient (61.21%) than strict families (13.46%). The outcomes infer the potential role of involvement, engagement, and influence from role models to raise awareness and understand the boundaries of socially acceptable behaviors and contributors to well-being. Another study found that strong religious beliefs integrated into society are a crucial protective factor in preventing adolescents from engaging in drug abuse [27]. Despite the firm norms and beliefs among the Saudi people and the existing legal probations against using alcohol and narcotic substances, a large proportion of Saudi residents still abuse drugs and alcohol [5]. 

The substances that the respondents commonly used for the first time were hashish (53%), alcohol (42%), and amphetamine (33%). A similar study conducted in Riyadh showed that alcohol, amphetamines, heroin, and cannabis were the most frequently abused substances in the community [22]. A study by Cambridge University found that cannabis is widely used by young adults [28]. Another case-control study at Albaha Psychiatric Hospital indicated that the most abused substances were amphetamine and cannabis at 87.7% and 70.49%, respectively [29]. Similarly, a study conducted in Jeddah at Alaml Hospital with 101 participants found that amphetamines and alcohol were the choices of drugs for initial substance use [6].

In our study, most respondents reported the reason for their first use of drugs to be teenagers’ curiosity (56%), followed by joy-seeking (47%). Their curiosity may have been strongly influenced by peer pressure, especially among teenagers. An excellent correlation can be observed between our findings and those of studies conducted in Jeddah. For instance, another research conducted in Jeddah claimed that the primary reasons for initial drug use were curiosity, peer group influence, traveling abroad, and psychiatric disturbances [6]. Similarly, another study with a sample size of 612 participants found that teenagers' curiosity was a risk factor for commencing drug use [2].

There is a possible correlation between early exposure and normalization of substance use and abuse in children. These exposures are largely due to a lack of awareness or guidance, leading to ease of influence, especially within the respective social groups, exacerbated by a lack of family involvement and engagement between parents and children. It has been shown that marriage and familial responsibility can mediate substance abuse, with parents serving as role models for their children. However, a family history of substance use can influence a predisposition towards substance use, especially among males, resulting in intergenerational use and abuse. Single people are more likely to experience progressive addiction without educational, religious, spousal, or family support from the ensuing stress and hardship of later life situations, leading people to resort to substance use. A similar study conducted on 1,391 adolescents indicated that the main risk factors for drug abuse were gender, age, low bonding with family, academic failure, and unstable marital status [30]. A relatively good socio-economic status can mediate the nature of substance use and abuse outcomes relative to the impact and implications of the prior variables discussed. Also significant from the data is that people in the Saudi culture generally get married during their early adulthood at around the age of 23, informed by the age range where the discrepancy between use and progression towards addiction is experienced.

Limitations

Some limitations of this study must be acknowledged. First, we used a self-reported questionnaire that cannot be independently verified and may be biased, as the participants may have sought social desirability while responding. Second, since the topic of substance use is sensitive in Saudi Arabia, it was not easy to connect with potential participants and find a convenient sample who had a substance use history. Therefore, the study sample size was not as large as would have been preferred to describe better and generalize the results. This may be because Saudi Arabia is an Islamic country that strictly prohibits the use of all types of illicit drugs and alcohol, and anxiety and fear of governmental laws and societal judgment could have been a factor preventing some individuals from participating in this study. Last, the cross-sectional design may have limited the detection of possible associations among the study variables. A qualitative design is suggested for future studies to triangulate the results regarding substance use in Saudi Arabia.

Recommendations for policy and research

The following recommendations are suggested to improve empirical evidence and raise awareness of the factors related to substance use toward prevention and improved health outcomes. First, we found that the risk of initial substance use started during school age and mostly with school friends. Young adults must be educated from adolescence about the harms of substance use engagement and its related risk factors and consequences. It might be useful to conduct drug abuse and awareness nursing campaign events in schools and public places, such as shopping malls, and to create trusted media platforms to raise awareness and knowledge surrounding the risk of drugs and minimize curiosity. Moreover, customized workshops for parents to raise awareness of the factors related to substance misuse are necessary because, based on our study results, marital status and parental education play major roles in minimizing the risk of initial drug use. Finally, similar studies must be conducted using mixed-method approaches and by recruiting larger sample sizes to enhance the accuracy of the findings and enrich the literature on initial exposure to substances and related socio-demographics in the Saudi Arabian context. ‏

## Conclusions

This study described the socio-demographic characteristics of initial exposure to substance use and its relation to substance abuse progression in Saudi Arabia. Some important factors that showed statistical differences with substance abuse progression were female gender, young adult groups (19‒23 years), school friends as company in drug use, father's educational level, and marital status. The study results indicated a strong statistical association between female gender and substance-use progression. Therefore, it is imperative to conduct gender-specific studies that compare substance abuse between gender categories while differentiating the risk factors that affect different gender groups. Further, longitudinal studies are necessary to determine the causality between substance use and different associated socio-demographic risk factors.

## Appendices

**Table 2:** Socio-demographics questionnaire: Supporting Information S1

Item	Answers
Gender	Male
	Female
Age	18-22
	22-26
	26-30
	More than 30
Family size when you first start using drugs/alcohol	3
	4
	5
	6 or more
Marital Status when you first start using drugs/alcohol	Single
	Married
	Separated
What was your educational status when you first start using drugs/alcohol?	Primary
	Middle
	High
	College
	Never educated
What was your parent's marital status when you first start using drugs/alcohol?	Together
	Separated
	Divorced
	Death of parent
How many wives your father had when you first start using drugs/alcohol?	0
	1
	2
	3
	4
How did you first know and used drugs/alcohol with?	Parents
	Siblings
	Cousins
	School friends
	Friends outside school
	Girlfriends or boyfriends
	Other
Father's educational level when you first start using drugs/alcohol	None
	School
	College
	Higher education
Mother's educational level when you first start using drugs/alcohol	None
	School
	College
	Higher education
What was the status of your father's occupation when you first start using drugs/alcohol?	Non employed
	Employed
	Retired
	Not applicable
What was the status of your mother’s occupation when you first start using drugs/alcohol?	Non employed
	Employed
	Retired
	Not applicable
What was your socioeconomic class when you first start using drugs/alcohol?	Poor
	Middle
	High middle
	Rich
How do you describe the religious status of your family when you first start using drugs/alcohol?	Strict
	In between of strict and lenient.
	Lenient
	Not religious
How do you describe the religious status of yourself when you first start using drugs/alcohol?	Strict
	In between of strict and lenient.
	Lenient
	Not religious
What is the name of the drug you used for the first time?	Xanax (benzodiazepine)
	Amphetamine (lajjah)
	Hashish
	Ecstasy
	Crystal myth
	Morphine
	Crack
	LSD
	Lyrica
	Heroin
	Cocaine
	Gat/Qat (*Catha edulis*)
	Shammah (chewing tobacco)
	Alcohol
	Others
What was your age when you first start using drugs/alcohol?	Younger than or 12
	13-18
	19-23 24-29
	30-35
	Older than 35
What was the first reason you start using drugs/alcohol for?	Teenagers curiosity
	Joy-seeking
	Psychological disorder
	Lack of knowledge about complications of drugs
	Positive attitude toward drug abuse
	Low self-confidence
	To eliminate shyness
	Parents’ divorce
	Lack of amusement facilities
	Disability in resolving routine problems
	Crowded family
	Having strict parents
	Presence of an addicted person in the family friends’ offer
	Family disputes
	Access to drugs
	Lack of access to consultation Centres
	Low cost of drugs
	Having free time
	Presence of an addicted person in residential/educational place
	Others
Do you still continue using drugs/alcohol?	Yes
	No
